# The blood pressure lowering effect of beetroot juice is impaired in periodontitis and recovered after periodontal treatment

**DOI:** 10.1038/s41522-024-00622-5

**Published:** 2025-01-09

**Authors:** Nydia Y. Sanchez-Orozco, Bob T. Rosier, Alondra Ruiz-Gutierrez, Fabiola Marquez-Sandoval, Alejandro Artacho, Lucrecia Carrera-Quintanar, Alex Mira

**Affiliations:** 1 PhD Program in Translational Nutrition Sciences, Department of Human Reproduction, Child Growth and Development, University Center of Health Sciences (CUCS), University of Guadalajara (UdeG), Guadalajara, Jalisco Mexico; 2 Department of Health and Genomics, FISABIO Foundation, Valencia, Spain; 3 Specialty of Periodontics, Department of Integral Dental Clinics, University Center of Health Sciences (CUCS), University of Guadalajara (UdeG), Guadalajara, Jalisco Mexico; 4 CIBER Center for Epidemiology and Public Health (CIBER-ESP), Madrid, Spain

**Keywords:** Microbiome, Plaque

## Abstract

We have previously demonstrated that subgingival levels of nitrate-reducing bacteria, as well as the in vitro salivary nitrate reduction capacity (NRC), were diminished in periodontitis patients, increasing after periodontal treatment. However, it remains unclear if an impaired NRC in periodontitis can affect systemic health. To determine this, the effect of nitrate-rich beetroot juice (BRJ) on blood pressure was determined in 15 periodontitis patients before and 70 days after periodontal treatment (i.e., professional mechanical plaque removal, oral hygiene instruction, and subgingival instrumentation), as well as in a healthy control group of 15 individuals. Additionally, subgingival and tongue samples were taken to analyse the bacterial composition with Illumina sequencing of the 16S rRNA gene. In healthy individuals, the systolic and diastolic blood pressure (SBP and DPB) decreased significantly (both *P* < 0.01) 90 min after BRJ intake, but not in periodontitis patients. However, after periodontal treatment, this blood pressure-lowering effect was recovered (*P* < 0.05 for SBP; *P* < 0.01 for DBP). Lower levels of salivary nitrate after identical doses of BRJ intake indicated a potentially higher NRC in healthy individuals (*P* < 0.05). Periodontitis-associated bacteria decreased in tongue and subgingival samples after periodontal treatment (*P* < 0.01). In contrast, nitrate-reducing bacteria were associated with health in both habitats, but increased only in subgingival plaque after periodontal treatment (*P* < 0.001). This is the first study showing that periodontitis could limit the blood-pressure lowering effects of nitrate reduction by the oral microbiota. We propose that an impaired NRC represents a potential link between periodontitis and systemic conditions, which should be confirmed in future randomized controlled trials. Future work should also aim to determine if nitrate prebiotic supplementation and/or tongue cleaning could improve the treatment of periodontitis and its associated comorbidities.

## Introduction

The oral microbiota normally consists of 100–200 bacterial species per individual of more than 700 oral species that have been identified in the oral cavity^1^. While there are clear differences in bacterial composition between individuals, certain functions are preserved, and this is known as functional redundancy^2^. One of these functions appears to be the reduction of nitrate to nitrite, which is conserved in most healthy individuals^3–5^. This biochemical reduction step, which human cells cannot do efficiently, is part of the nitrate-nitrite-nitric oxide pathway^6^. This pathway is a fascinating example of symbiosis between the human body and the oral microbiota that, after dietary nitrate ingestion, results in an increase of systemic nitric oxide availability. Nitric oxide is an important signalling molecule and vasodilator of the human body that can improve the cardiometabolic and oral health of the host^6–8^.

The steps of the nitrate-nitrite-nitric oxide pathway have been previously discussed in detail by different groups^6,7,9,10^. Briefly, when consuming nitrate-rich vegetables such as leafy greens or beetroots, nitrate is absorbed into the bloodstream through the gastrointestinal tract, leading to an increase in plasma nitrate levels. The salivary glands then actively concentrate this nitrate into the saliva. While human cells cannot metabolize nitrate efficiently, certain oral bacteria can reduce nitrate to nitrite and further into nitric oxide and other denitrification products^9^. The nitrate recycling by the salivary glands significantly increases plasma nitrate and nitrite levels for several hours after nitrate intake, thereby enhancing nitric oxide bioavailability^11^ Among the most studied outcomes of consuming nitrate-rich vegetables, particularly beetroot juice, is the acute reduction in blood pressure observed 1–3 h post-ingestion^12^. Additionally, nitrate intake has been shown to improve endothelial function, enhance sport performance, and potentially exert antidiabetic effects^8^.

It has been found that sterilizing and inhibiting a significant proportion of the oral microbiota with chlorhexidine can impair the nitrate reduction capacity (NRC) of the oral microbiota. For example, in fasting individuals, chlorhexidine can lead to a decrease in plasma nitrite and increase in blood pressure^13^. Chlorhexidine can also interfere with (beneficial) physiological changes after nitrate intake, such as post-exercise hypotension and skeletal muscle oxygenation^14,15^.

Similar to the effect of antiseptics, other conditions that lead to lower levels or an impaired activity of nitrate-reducing bacteria (e.g., representatives of *Rothia*, *Neisseria*, *Acinomyces*, *Veillonella*, *Kingella* and *Propionibacterium*) could affect the NRC of the oral microbiota. In a previous study, we showed that the total level of nitrate-reducing bacteria decreases in subgingival plaque of patients with periodontitis and that their salivary NRC (determined in vitro) was impaired^5^. However, after periodontal treatment (i.e., professional mechanical plaque removal, oral hygiene instruction, and subgingival instrumentation), a reduction in inflammation^16^ was accompanied by an increase in nitrate-reducing bacteria and a recovery of the salivary NRC to healthy levels^5^. Nevertheless, there are no clinical studies that have tested the effect of nitrate intake in individuals with different oral health conditions in vivo. The aim of this clinical study was thus to test the effect of periodontitis, before and after periodontal treatment, on the blood-pressure lowering effect of beetroot juice.

Moreover, existing evidence that nitrate-reducing bacteria decrease in periodontitis is limited to subgingival plaque. However, the microbial community on the tongue harbours the largest proportion of oral bacteria and has classically been considered most important for the nitrate-nitrite-nitric oxide pathway^17^. A second aim was therefore to test how nitrate-reducing bacteria on the tongue were affected by periodontitis.

For this, 15 periodontally healthy individuals and 15 periodontitis patients before and after periodontal treatment consumed beetroot juice. The effects on blood pressure and salivary nitrate and nitrite were determined. Additionally, the composition of the subgingival and tongue microbiota was determined by sequencing of the 16S rRNA gene to establish the effect of periodontitis and periodontal treatment on these communities. Specifically, differences in nitrate-reducing bacteria and periodontitis-associated species were assessed.

## Methods

### Study population, treatment, and clinical examination

This clinical study was conducted under the Declaration of Helsinki and the ethical guidelines developed by CIOMS (Council for International Organizations of Medical Sciences). All the subjects before enrollment to the study provided signed written informed consent, and the protocol was approved by the Research, Ethical and Biosafety Committees of the University Center of Health Sciences, University of Guadalajara (Code CI-05722 CUCS-UdeG), based on the international ethical guidelines. A total of 30 adult subjects were enrolled with an age range between 25 and 65 years (all descriptive data are presented in Table 1).

**Table 1:** Baseline characteristics of study participants (averages ± standard deviations)

	All (*n* = 30)	Healthy (*n* = 15)	Periodontitis (*n* = 15)	*p*
**Age (yrs)**	45.73 ± 9.35	44.46 ± 8.313	47.2 ± 10.798	0.464^a^
**Male, female (%)**	40, 60	53, 47	27, 73	0.264^b^
**Weight (kg)**	70.93 ± 14.15	72.41 ± 16.76	69.18 ± 11.53	0.554^a^
**Waist (cm)**	85.64 ± 10.42	85.10 ± 12.92	86.04 ± 8.09	0.815^a^
**BMI (kg/m**^**2**^**)**	26.16 ± 4.22	25.44 ± 3.82	26.99 ± 4.85	0.369^a^
**SBP (mmHg)**	119.35 ± 13.46	118.61 ± 15.84	120.09 ± 11.09	0.949^a^
**DBP (mmHg)**	72.07 ± 8.31	72.66 ± 7.37	76.02 ± 8.03	0.264^a^
**HR (bpm)**	73.57 ± 8.37	74.28 ± 8.99	72.85 ± 7.93	0.507^a^
**Total calorie intake (kcal)**^**c**^	2,394.35 ± 1,032.58	2,057.10 ± 767.80	2,709.13 ± 1,168.38	0.090^a^
**Dietary fiber (g)**^**c**^	42.70 ± 28.18	34.24 ± 13.97	50.04 ± 35.21	0.142^a^
**Sodium (mg)**^**c**^	3,847.38 ± 4,360.60	3,329.12 ± 2,774.98	4,296.50 ± 5,441.09	0.568^a^
**Calcium (mg)**^**c**^	1,114.12 ± 601.46	1,019.24 ± 629.56	1,196.36 ± 585.10	0.447^a^
**Potassium (mg)**^**c**^	4,368.78 ± 2,210.24	3,787.90 ± 1,448.05	4,872.22 ± 2,653.15	0.201^a^
**Alcohol (g)**^**c**^	4.33 ± 4.93	5.12 ± 5.53	3.65 ± 4.42	0.443^a^

^a^ comparisons between baseline healthy *vs* periodontitis (unpaired *t* test)

^b^ comparison between categorical variables (chi-square test)

^c^ Dietary parameters were determined using a food frequency questionnaire, and the values represent daily intake

*BMI* Body mass index, *SBP* systolic blood pressure, *DBP* diastolic blood pressure, *HR* heart rate.

Individuals were selected based on their periodontal health and classified into two groups: periodontally healthy individuals (*n* = 15) and patients with periodontitis (*n* = 15). The diagnosis of periodontitis followed the 2017 classification of periodontal diseases by Pappanou et al.^18^. The periodontally healthy group included individuals with no radiographic evidence of periodontal bone loss, periodontal probing depths (PPD) ≤3 mm, and bleeding on probing (BoP) ≤10%, without clinical attachment loss (CAL)^19^. Periodontitis patients who required periodontal treatment were recruited at the periodontics specialty clinic at the University of Guadalajara in Mexico. The eligible criteria for periodontitis were interdental CAL ≥ 3 mm of two or more non-adjacent teeth, PPD ≥ 4 mm, and radiographic evidence of periodontal bone loss^20^. Study subjects were excluded they had previously been diagnosed with diabetes, cardiovascular disease, rheumatoid arthritis, a known or suspected risk of tuberculosis, hepatitis B or HIV infections, a history of bleeding diathesis, or any other systemic disease or conditions (like pregnant women or women in lactation) that could alter the course of periodontal disease. Additionally, individuals with a smoking habit, those who had taken antibiotics, anti-inflammatory drugs or oral antiseptics in the last 3 months, and periodontal patients who had received periodontal therapy in the preceding six months were excluded.

Patients received periodontal treatment, including professional mechanical plaque removal (PMPR) and subgingival instrumentation at all sites requiring this^21^ (previously referred to as non-surgical periodontal treatment, NSPT). Both ultrasonic and manual instrumentation were used throughout the study, which were previously reported to lead to similar microbiological and clinical outcomes^22^. The periodontal treatment was conducted by an experienced periodontist in two visits (quadrant 1 and 4 during visit 1 and quadrant 2 and 3 during visit 2). It is important to note that antibiotics or chlorhexidine and other antiseptics were not used or prescribed during the study. As part of the periodontal treatment, patients received comprehensive instructions and regular monitoring of their oral care at home, provided by the periodontist. This guidance included proper brushing techniques, interdental hygiene, and plaque control. No additional instructions were given to modify other aspects of their lifestyles. Full-mouth periodontal parameters were assessed before the treatment (baseline, BL) and again 70 days following the treatment (D70). These included bleeding on probing (BoP), plaque or calcus scores (PoC), periodontal pocket depths (PPD), and, clinical attachment level (CAL), which were divided by the total number of sites to give average full-mouth scores.

In the clinical evaluation, measurements of blood pressure, salivary flow, and saliva collection were taken before and after 1.5 hours after ingestion of a beet juice with a known concentration of nitrate, at both BL and D70. Patients were asked to abstain from eating nitrate-rich vegetables and processed meats for 24 h before the BL and D70 visits. Additionally, they were asked not to consume any water or food 4 h before each visit. Anthropometric measurements followed the protocol of the International Society for the Advancement of Kineanthropometry (ISAK)^23^. Weight, height, and waist circumference were recorded, and the body mass index (BMI) (kg/m²) was calculated. Additionally, a food frequency questionnaire (FFQ) was used to determine habitual dietary intake and alcohol consumption, and a photographic album of Mexican foods helped estimate portion sizes. Both tools were validated for the Mexican population^24,25^.

### Sample collection and processing

Samples were collected from healthy individuals at one-time point and from periodontitis patients before (BL) and 70 days after (D70) treatment. Saliva was collected using the passive drool method^26^ and the flow rate was measured for 5 minutes. To homogenize sample collection across donors, subgingival plaque was collected with Gracey curettes from a defined set of the mesial surface teeth if present (numbers 16-14-11-21-24-26-36-34-31-41-44-46 according to FDI nomenclature) and tongue coating samples were collected with a spatula by scraping the most posterior part of the tongue^27^. The samples were stored in tubes with DNA shield (DNA/RNA Shield™, Zymo Research) for preservation of the genetic material. All samples were stored at -80 °C until analysis or following procedure.

### Blood pressure measurements

Individuals were requested to sit down in a quiet room for 15 min. It was ensured that the subject was seated with a straight back, both feet on the floor, well supported and the arm resting on a flat surface. Blood pressure was measured by an OMRON HEM-742 digital sphygmomanometer on each arm. This was repeated three times and averages were taken^28^. The blood pressure measurements were taken on at least one day before the periodontal treatment to limit the effect of dental anxiety.

### Pre- and post-beetroot juice measurements

A large batch of beetroot juice was obtained from beetroots at the Food Science Laboratory (CUCS, University of Guadalajara, Mexico) and bottled in individual flasks with 350 ml beetroot juice (4.23 g/L nitrate) that were frozen at −20 °C and only defrosted before consumption. After the clinical evaluation and sample collection, individuals would take one bottle of beetroot juice. One and a half hour after beetroot juice intake, another saliva sample was taken and the blood pressure was measured again. This time was based on the observation that blood pressure-lowering effects start around 1 h after intake^29^.

### Quantification of nitrate and nitrite in saliva

For nitrate and nitrite measurements in saliva, the RQflex 10 Reflectoquant (Merck Millipore, Burlington, Massachusetts, USA) reflectometer was used as described by Rosier et al.^3^. The test strips (Reflectoquant, Merck Millipore) for nitrate had a range of 3–90 mg/L and the strips for nitrite a range of 0.5–25 mg/L. The saliva before beetroot juice was used directly or diluted 2–10 times with water, while the saliva 2 h after beetroot juice was diluted 2–20 times. Fifteen µL of (diluted) saliva was added to each of the two reactive patches on a strip and excess liquid was removed by tipping the side of the strip on a tissue. Before nitrate measurements, the diluted supernatants were treated with amidosulfuric acid (Sigma-Aldrich) based on the manufacturer’s instructions. For this, 35 µL of diluted supernatant was mixed with 1.5 µL amidosulfuric acid solution (10%).

### Bacterial 16S rRNA sequencing

DNA was extracted with the Quick-DNA TM Fungal/Bacterial Miniprep kit (Zymo Research, USA), according to the manufacturer’s instructions and was stored at −20 °C until used for sequencing. DNA concentrations were measured using a QubitTM 3 Fluorometer (ThermoScientific). An Illumina amplicon library was prepared following the 16S rRNA gene Metagenomic Sequencing library preparation Illumina protocol (Part #15,044,223 Rev. A), using gene specific 16 S amplicon PCR primers for the V3 and V4 region, resulting in amplicons of ~460 bp (F: 5′ TCGTCGGCAGCGTCAGATGTGTATAAGAGACAGCCTACGGGNG GCWGCAG 3′; R: 5′ GTCTCGTGGGCTCGGAGATGTGTATAAGAGACAGGACTACHVGGGTATCTAATCC 3′). Following amplification, DNA was sequenced with an Illumina MiSeq Sequencer according to the manufacturer’s instructions using the 2 × 300 base paired-ends protocol. For taxonomic classification, an amplicon sequence variant (ASV) table was obtained using the DADA2 pipeline in R^30^. Taxonomy was assigned to each ASV using the DADA2 classifier by comparison to the SILVA database^31^. The ASVs with an assigned genus but without an exact species were aligned using the BLASTn tool (v2.10.0+) against the Silva database with a minimum identity threshold of 97%.

### Data analysis

The clinical and physiological parameters were analyzed using GraphPad (v9.01). The distribution of all clinical and physiological data was checked to determine the statistical test. If normally distributed, a paired *t* test was used for matched comparisons (periodontitis-BL versus periodontitis-D70) and an unpaired *t* test for independent groups comparisons (health versus periodontitis). If one of the groups was not normally distributed, Wilcoxon’s test was used for paired comparisons and the Mann-Whitney U test for unpaired comparisons.

For analysis of the subgingival plaque and tongue microbiome, R programming language (v4.2.2) was used for statistical computing^32^. Species or genera were removed if they were not present in at least 60% of the samples in one of the groups (periodontitis-BL, periodontitis-D70, or health) with an abundance of 10 times the smallest percentage above zero. The filtered abundances were used to determine the Alpha-diversity indexes (Shannon, ACE, Chao1) and all data visualization.

To compare species or genera between health, periodontitis-BL, and periodontitis-D70, the filtered abundances were first transformed with the standard compositional data analysis technique, ANCOM-BC^33^. Negative values were then removed by adding the lowest negative value to all transformed abundances. Genera and species were analysed separately and as groups of species, including nitrite-producing species, confirmed nitrate-reducing species, periodontitis-associated species and red complex as done previously^5^. The disease-associated species were based on the Socransky orange and red complexes^34^ and a systematic review^35^. Nitrite-producing bacteria are those that have been shown to produce nitrite in the presence of nitrate by detection of nitrite (which could also have been produced by other pathways), while confirmed nitrate reducing bacteria have been shown to reduce nitrate to nitrite by physiological measurements of nitrate^7^.

For univariate analyses, Wilcoxon signed rank tests (i.e., wilcox.test function of stats library of R) was performed to test the differences in genera and species between baseline and day 70, corrected for multiple comparisons using the Benjamini–Hochberg false discovery rate (FDR) of 5%^36^. Thus, only adjusted *p*-values are reported for taxonomic comparisons. For multivariant analysis, a Constrained Correspondence Analysis (CCA) was performed, including a CCA *p*-value and Adonis test (Permutational Multivariate Analysis of Variance Using Distance Matrices) *p*-value, which are all part of the Vegan library of R^37^.

All comparisons between periodontitis-BL and periodontitis-D70 were paired, while the comparisons between periodontitis (BL or D70) and health were unpaired. All graphs were assembled using GraphPad PRISM (v9.01) or R.

Correlations between different genera and species or between taxa and other parameters were determined with Spearman’s rho, along with associated adjusted *p*-value using the cor.test function.

## Results

### Study participants and clinical parameters

Patients who required periodontal treatment (*n* = 15) and periodontally healthy patients (*n* = 15) were recruited. The average age of the participants was 45.73 (± 9.35) years, 60% were female, and no significant differences were found at baseline between descriptive variables, dietary parameters, or blood pressure between the two groups (Table 1). Based on the 2017 classification, most participants had generalized periodontitis stage 3 or 4^18^. Specifically, they were classified as stage 3 grade C (33%), stage 3 grade B (33%), stage 4 grade C (20%), stage 3 grade A (7%), or stage 2 grade B (7%). As expected, there was a clear significant difference in periodontal health parameters between the groups (*p* < 0.0001 in all cases) (Table 2). All clinical and physiological parameters can be found in the Supplementary Datasheet.

**Table 2:** Baseline and post-treatment periodontal parameters (full-mouth averages ± standard deviations)

	Healthy		Periodontitis	p^*^
**PD (mm)**	2.34 ± 0.47	BL	3.49 ± 0.43	< 0.0001
		D70	2.80 ± 0.47	
		***p***^***+***^	0.004	
**CAL (mm)**	0.29 ± 0.52	BL	3.31 ± 0.73	< 0.0001
		D70	2.83 ± 0.65	
		***p***^***+***^	0.005	
**BoP (%)**	11.10 ± 7.00	BL	63.09 ± 28.40	< 0.0001
		D70	36.41 ± 15.21	
		***p***^***+***^	0.017	
**PoC (%)**	12.87 ± 20.89	BL	52.50 ± 27.08	< 0.0001
		D70	34.16 ± 22.98	
		***p***^***+***^	0.077	

p^*^: Comparisons between healthy vs periodontitis at baseline (BL) (unpaired *t* test)

***p***^***+***^: Comparisons between periodontitis at BL vs 70 days after treatment (D70) (paired *t* test)

*PD* Probing on depth, *CAL* clinical attachment level, *BOP* bleeding on probe, *PoC* plaque or calculus

Seventy days after periodontal treatment, patients with periodontitis showed significant improvements in PD, CAL, and BoP (all *p* < 0.05), but not in the percentage of teeth with visible plaque or calculus (*p* = 0.08) (Table 2).

### Blood pressure before and after beetroot juice ingestion

**Fig. 1:**
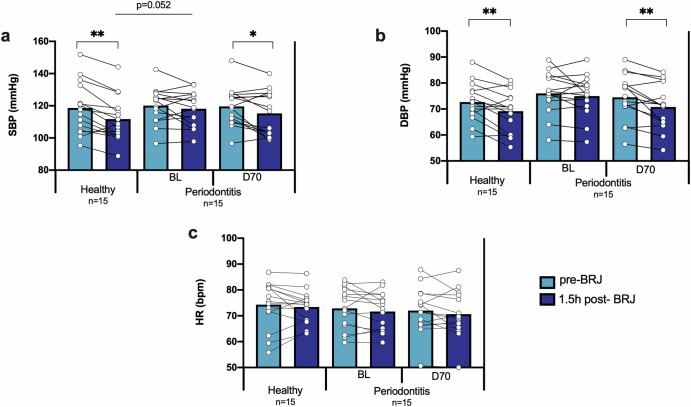
Blood pressure pre- and post-beetroot juice. The effect of beetroot juice on the systolic blood pressure (**a**), diastolic blood pressure (**b**), and heart rate (**c**) was determined in 15 healthy individuals and 15 periodontitis patients before (BL) and 70-days after (D70) periodontal treatment. The light blue bars are the parameters before beetroot juice (pre-BRJ) intake and the dark blue bars 1.5 hours after beetroot juice intake (1.5 h post-BRJ). SBP: systolic blood pressure. DBP: diastolic blood pressure. HR: heart rate. BRJ: beetroot juice. *p < 0.05 and **p < 0.01 according to a paired or unpaired *t*-test for parametric comparisons.

The blood pressure of all participants was assessed before and 1.5 h after drinking beetroot juice (BRJ). In periodontally healthy patients the SBP and DBP decreased significantly after juice intake, while in patients with periodontitis this effect was not observed (Fig. 1). However, 70 days after receiving the periodontal treatment, the blood pressure lowering effect was significant (*p* < 0.05 for SBP and *p* < 0.01 for DBP). In relation to heart rate, the intake of BRJ did not cause a significant change in any of the groups, as expected.

### Salivary quantifications before and after beetroot juice consumption

**Fig. 2:**
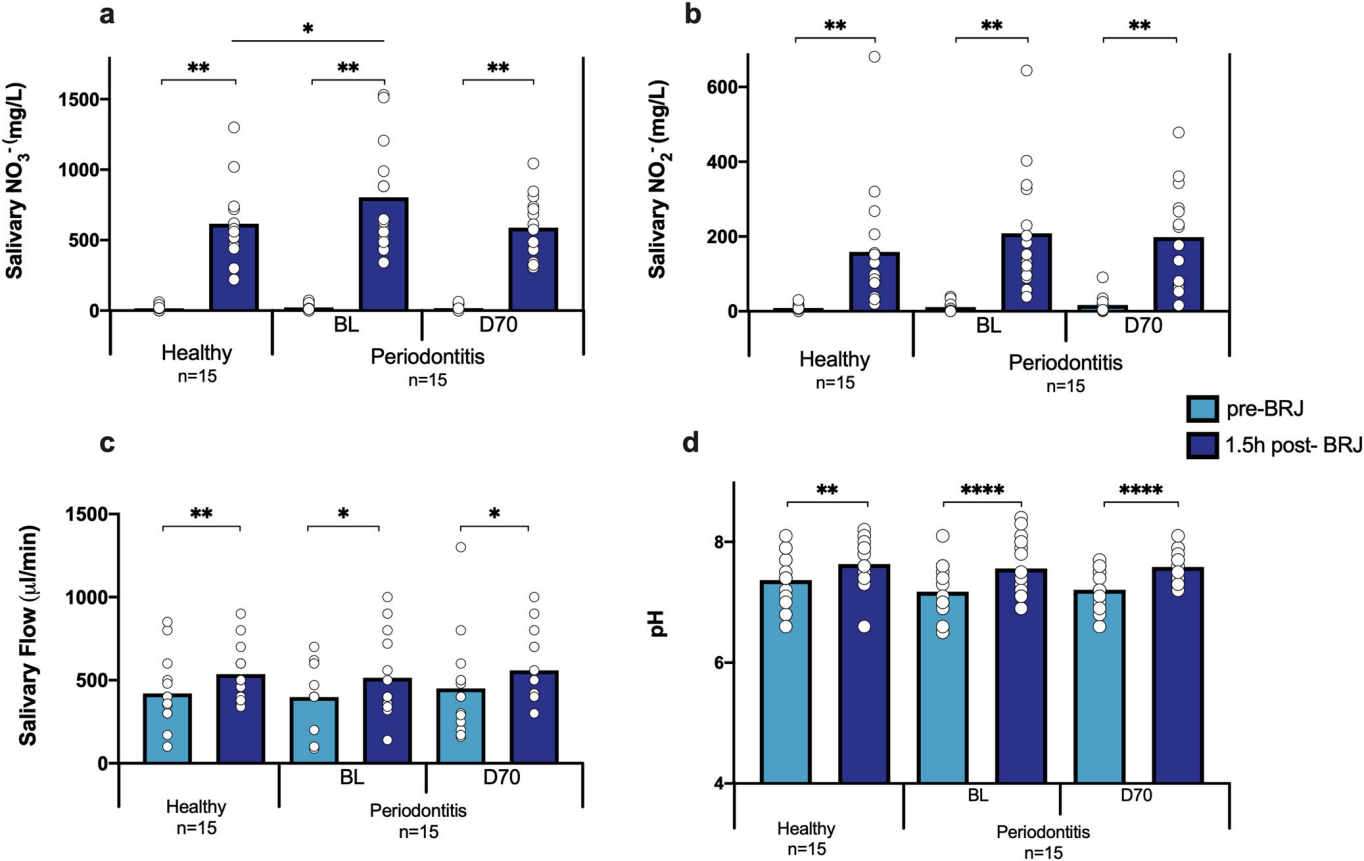
Comparison of measurements before and 1.5 h after BRJ consumption. The effect of beetroot juice on salivary nitrate (**a**) and nitrite (**b**), salivary flow (**c**) and pH (**d**) was determined in 15 healthy individuals and 15 periodontitis patients before (BL) and 70-days after (D70) periodontal treatment. The light blue bars are the parameters before beetroot juice (pre-BRJ) intake and the dark blue bars 1.5 h after beetroot juice intake (1.5 h post-BRJ). NO_3_^-^: nitrate. NO_2_^-^: nitrite. μl: microliters. Min: minute. Mg: milligrams. L: liter. Statistics: *p < 0.05 and **p < 0.01 according to an unpaired *t*-test or paired *t*-tes*t* for parametric comparisons and a Wilcoxon test for nonparametric comparisons.

The levels of nitrate and nitrite were quantified in saliva and an increase of both molecules was observed in all groups after beetroot juice consumption (all *p* < 0.01, Fig. 2a and b). However, a higher amount of post-beetroot juice salivary nitrate was detected in periodontitis patients at baseline compared to healthy individuals (*p* < 0.05) (Fig. 2a). Taking into account that the nitrate dose was identical, this indicates that more nitrate was reduced by healthy individuals. After periodontal treatment, the salivary nitrate levels after beetroot juice intake were not significantly different from healthy individuals. The salivary flow before and after beetroot juice was also determined (Fig. 2c). Remarkably, the beetroot juice increased the salivary flow significantly in all participants (Fig. 2c). Specifically, the salivary flow increased 115.33 ± 119.27 µl/min in healthy individuals (*p* < 0.01), 101.57 ± 157.27 µl/min in periodontitis patients at BL (*p* < 0.05) and 108.66 ± 242.54 µl/min in periodontitis patients at D70. Additionally, the beetroot juice increased the salivary pH in all groups (all *p* < 0.01) (Fig. 2d).

### Microbiome of subgingival plaque and tongue

The bacterial composition of subgingival plaque samples and tongue coating samples was determined through 16S rRNA gene Illumina sequencing. Three subgingival samples (out of 45 samples) and 2 tongue samples (out of 45) did not contain DNA after the extraction procedure. Additionally, 1 subgingival sample and 1 tongue sample were removed due to a low number of reads ( < 5000 reads). This led to a total number of 41 subgingival samples with an average of 157,697 reads (range: 90,063- 267,861 reads) and 42 tongue samples with an average of 152,488 reads (range: 75,836- 429,161 reads) per sample. The final sample size for the microbiota analyses was 13 healthy individuals and 14 periodontitis patients (with both BL and D70) for the tongue samples, and 13 healthy individuals and 13 periodontitis patients (with both BL and D70) for the subgingival plaque samples.

**Fig. 3:**
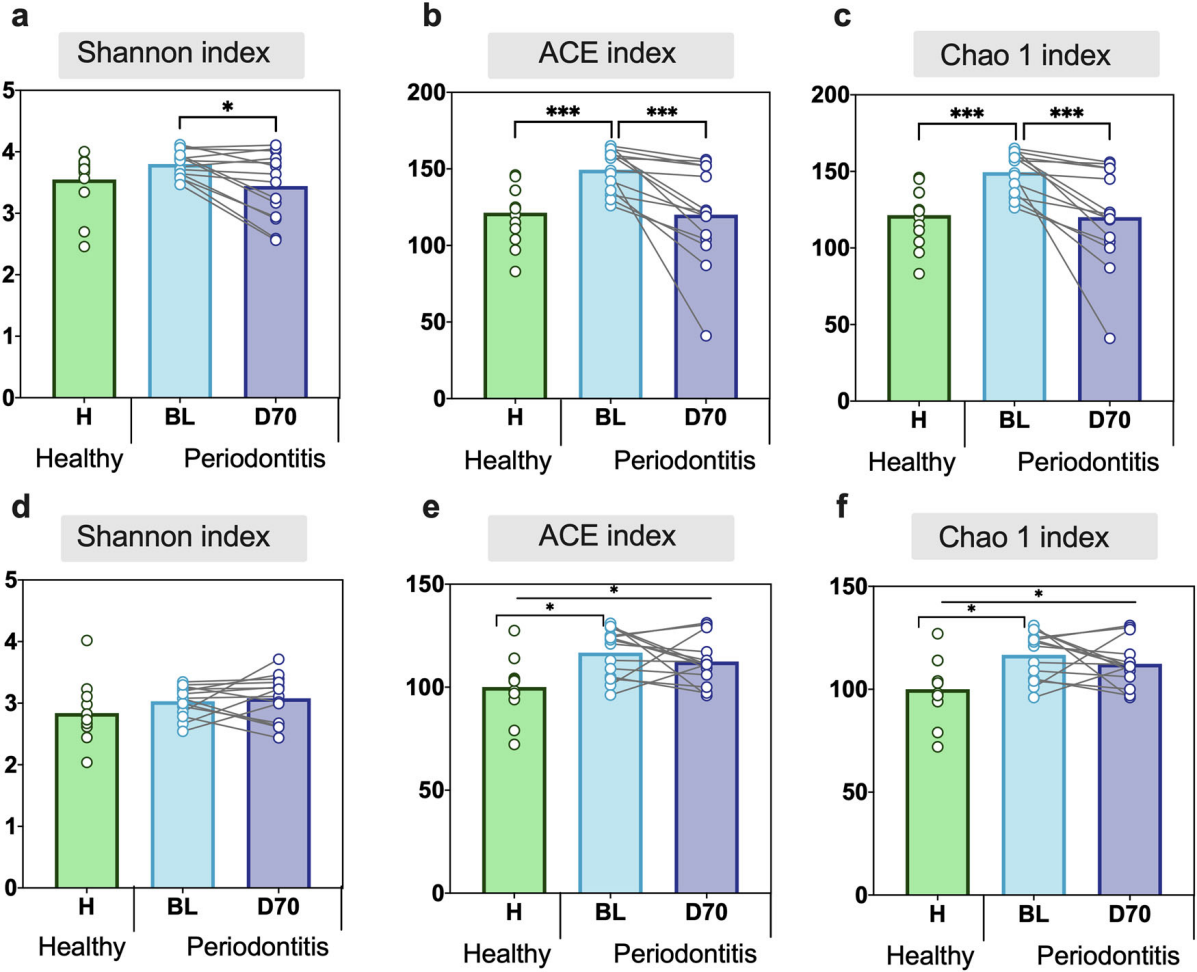
Alpha diversity of subgingival plaque and tongue samples at the species level. Comparing Shannon, ACE and Chao1 indexes in healthy individuals (green bars) and periodontitis patients at baseline (BL, light blue bars) and post-treatment (D70, dark blue bars). The alpha diversity of subgingival plaque (**a**–**c**) and tongue (**d**–**f**) samples is plotted. Statistics according to an unpaired or paired *t*-test, *p < 0.05 and **p < 0.01, ***p < 0.001.

When comparing subgingival plaque alpha-diversity between health and periodontitis at the species level, a significant difference was observed when using both the ACE (Fig. 3b) and Chao1 (Fig. 3c) indices (*p* < 0.001 in both cases), with periodontal patients showing higher bacterial richness. When evaluating the change in periodontitis patients after periodontal treatment (BL vs. D70), it was found that there was a significant decrease in the alpha-diversity indexes (all *p* < 0.05, Fig. 3a–c). In contrast, no significant differences were observed when comparing the alpha-diversity between healthy controls and post-treatment patients (Fig. 3a–c).

When analyzing the changes in tongue coating samples, it was observed that bacterial richness was significantly lower in healthy controls compared to periodontitis patients at baseline (H vs BL) when evaluated with the ACE and Chao1 indexes (Fig. 3e, f). In contrast to subgingival plaque, no significant effect of the periodontal treatment on tongue microbiota alpha-diversity was observed (Fig. 3D–F). As a consequence of this, the estimated number of species in the tongue remained significantly different between periodontally healthy individuals and periodontitis patients after treatment (H vs D70) when using the ACE and Chao1 indices (*p* < 0.05) (Fig. 3e-f).

**Fig. 4:**
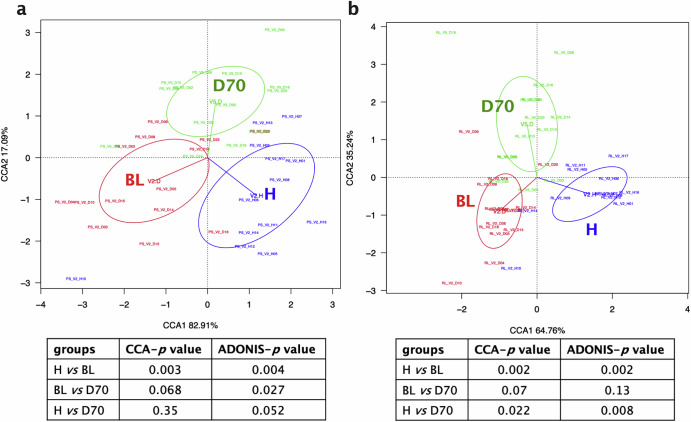
Beta diversity of subgingival plaque and tongue samples at the species level. Canonical Correspondence Analysis (CCA) comparing the bacterial composition of healthy individuals (blue) and periodontitis patients at baseline (BL, red) and 70 days after treatment (D70, green) at the species taxonomic level, as revealed by 16S rRNA gene Illumina sequencing. The CCA plots of subgingival plaque (**a**) and tongue (**b**) samples are shown. Tables below show comparisons between groups and CCA and Adonis *p* values.

Similar to the changes observed in alpha-diversity, differences between healthy controls and periodontitis patients in bacterial composition, as well as changes after periodontal treatment were observed at the species level in both subgingival plaque and tongue samples (Fig. 4). Specifically, the CCA plots show a change in subgingival plaque bacterial composition between health and periodontitis at baseline (BL) (Adonis *p* < 0.01), as well as a significant difference between BL and D70 (Adonis *p* < 0.05). The CCA and Adonis *p*-values between healthy individuals and periodontitis patients 70 days after treatment were not significant (CCA *p* = 0.35, Adonis *p* = 0.052) (Fig. 4a).

The CCA and Adonis results of the tongue community was also in line with the alpha-diversity results. There were significant differences when comparing healthy individuals with periodontitis patients at baseline (Adonis *p* < 0.01) and 70 days post-treatment (*p* < 0.01), but there were no significant differences when comparing periodontitis patients before and after treatment (*p* = 0.13) (Fig. 4b).

**Fig. 5:**
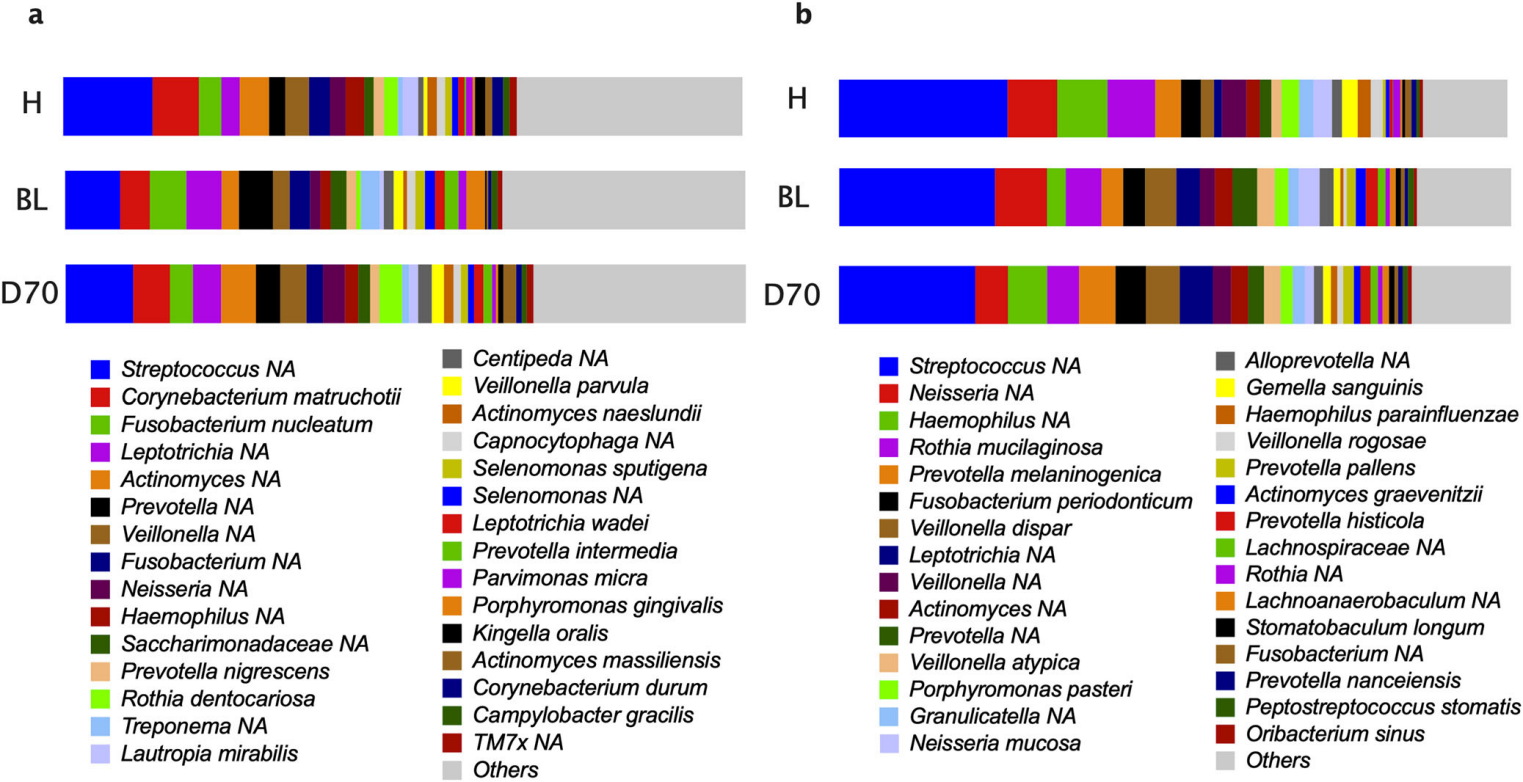
Relative bacterial abundances in subgingival plaque and tongue coating. Bar graphs show the top 30 most abundant species in healthy individuals (H) and periodontitis patients before (BL) and after periodontal treatment (D70). Relative abundances are shown in subgingival (**a**) and tongue (**b**) samples.

The most abundant bacteria in the subgingival plaque of all groups were unclassified *Streptococcus*. In health, these were followed by *Corynebacterium matruchotii*, unclassified *Actinomyces*, unclassified *Veillonella* and *Fusobacterium nucleatum*. In periodontitis before treatment (BL) these were followed by *Fusobacterium nucleatum*, unclassified *Leptotrichia*, unclassified *Prevotella* and *Corynebacterium matruchotii*, while after treatment they were followed by *Corynebacterium matruchotii*, unclassified *Actinomyces*, unclassified *Leptotrichia* and unclassified *Veillonella* (Fig. 5a).

In the tongue coating samples, the most abundant species were unclassified *Streptococcus*, unclassified *Haemophilus*, unclassified *Neisseria, Rothia mucilaginosa* and *Prevotella melaninogenic*a in health. In periodontitis before treatment (BL) these were unclassified *Streptococcus*, unclassified *Neisseria, Rothia mucilaginosa, Veillonella dispar* and unclassified *Prevotella* while after treatment these were unclassified *Streptococcus*, unclassified *Haemophilus, Prevotella melaninogenic*a, *Veillonella dispar* and unclassified *Leptotrichia* (Fig. 5b).

The bacterial composition at the genus level can be found in Supplementary Fig. 1. When grouping all individuals, the most abundant genera in subgingival plaque were *Streptococcus, Prevotella, Fusobacterium, Actinomyces* and *Corynebacterium*, while in the tongue coating were *Streptococcus, Prevotella, Veillonella, Neisseria* and *Haemophilus*.

**Fig. 6:**
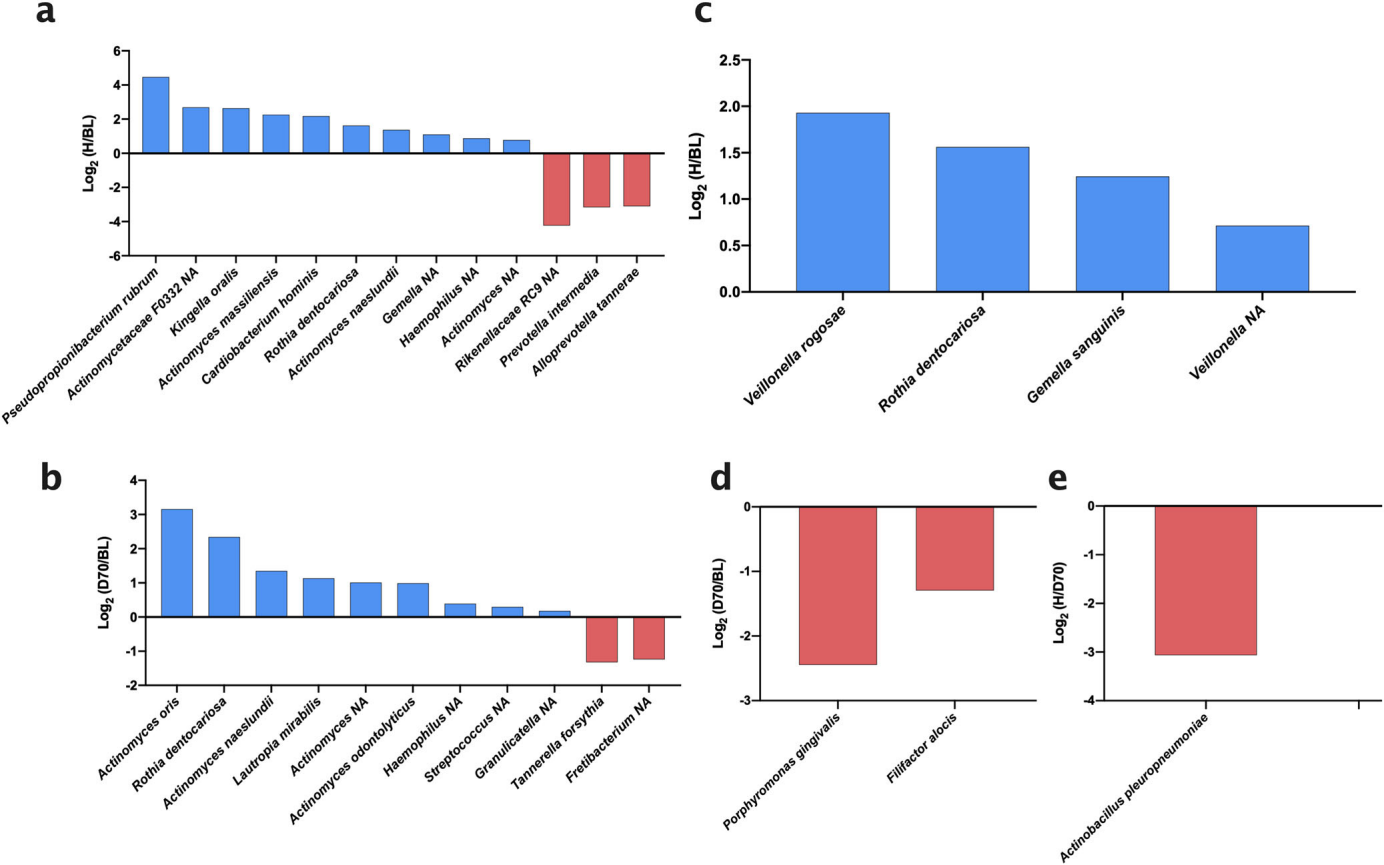
Log_2_ fold changes of all significantly different species in subgingival plaque and tongue samples. Bar graphs show comparisons between healthy individuals (H) and periodontitis patients at baseline (BL) and 70 days after treatment (D70). The groups that are compared are H and BL (**a**), and D70 and BL (**b**) in subgingival plaque, as well as H and BL (**c**), D70 and BL (**d**), and **h** and D70 (**e**) in tongue coating. Blue bars (positive values) indicate species which increased significantly after treatment or are more abundant in health; red bars (negative values) indicate species which significantly decreased after treatment or are more abundant in disease. To calculate the ratios, relative abundances were used. All plotted organisms were significantly different (adjusted *p*-values < 0.05) between two groups after standardization of the compositional data by ANCOM-BC. Organisms marked with NA indicate that they could not be assigned at the species level.

When comparing bacterial species in subgingival plaque between healthy controls and baseline periodontal patients, 13 significant differences were identified, of which 10 species were higher in health (*Pseudopropionibacterium propionicum*, unclassified *Actinomycetaceae F0332, Kingella oralis, Actinomyces massiliensis, Cardiobacterium hominis, Rothia dentocariosa, Actinomyces naeslundii*, unclassified *Gemella*, unclassified *Haemophilus*, and unclassified *Actinomyces*) and 3 were higher in baseline periodontitis (unclassified *Rikenellaceae RC9, Prevotella intermedia, Alloprevotella tannarae)* (Fig. 6a). When comparing periodontitis before (BL) and after (D70) treatment, we found 11 significant changes. Specifically, nine species increased post-treatment (*Actinomyces oris, Rothia dentocariosa, Actinomyces naeslundii, Lautropia mirabilis*, unclassified *Actinomyces, Actinomyces odontolyticus*, unclassified *Haemophilus* and unclassified *Streptococcus)* and two decreased (*Tannerella forsythia* and unclassified *Fretibacterium*) (Fig. 6b). It must be underlined that when comparing health and periodontitis after treatment, no significant changes were identified, in line with the diversity index, CCA and Adonis results.

When evaluating significant changes in tongue species, there were significant differences between healthy individuals and patients with periodontitis at baseline. Specifically, we found 4 species that were higher in healthy individuals (*Veillonella rogosae*, *Rothia dentocariosa, Gemella sanguinis* and unclassified *Veillonella)* (Fig. 6c). Also, there were significant changes in tongue species after periodontal treatment, including two periodontal pathogenic species that decreased (*Porphyromonas gingivalis* and *Filifactor alocis*) (Fig. 6d). When comparing patients on day 70 post-treatment versus healthy patients, there is only one significantly lower species (*Actinobacillus pleuropneumoniae*) (Fig. 6e).

**Fig. 7:**
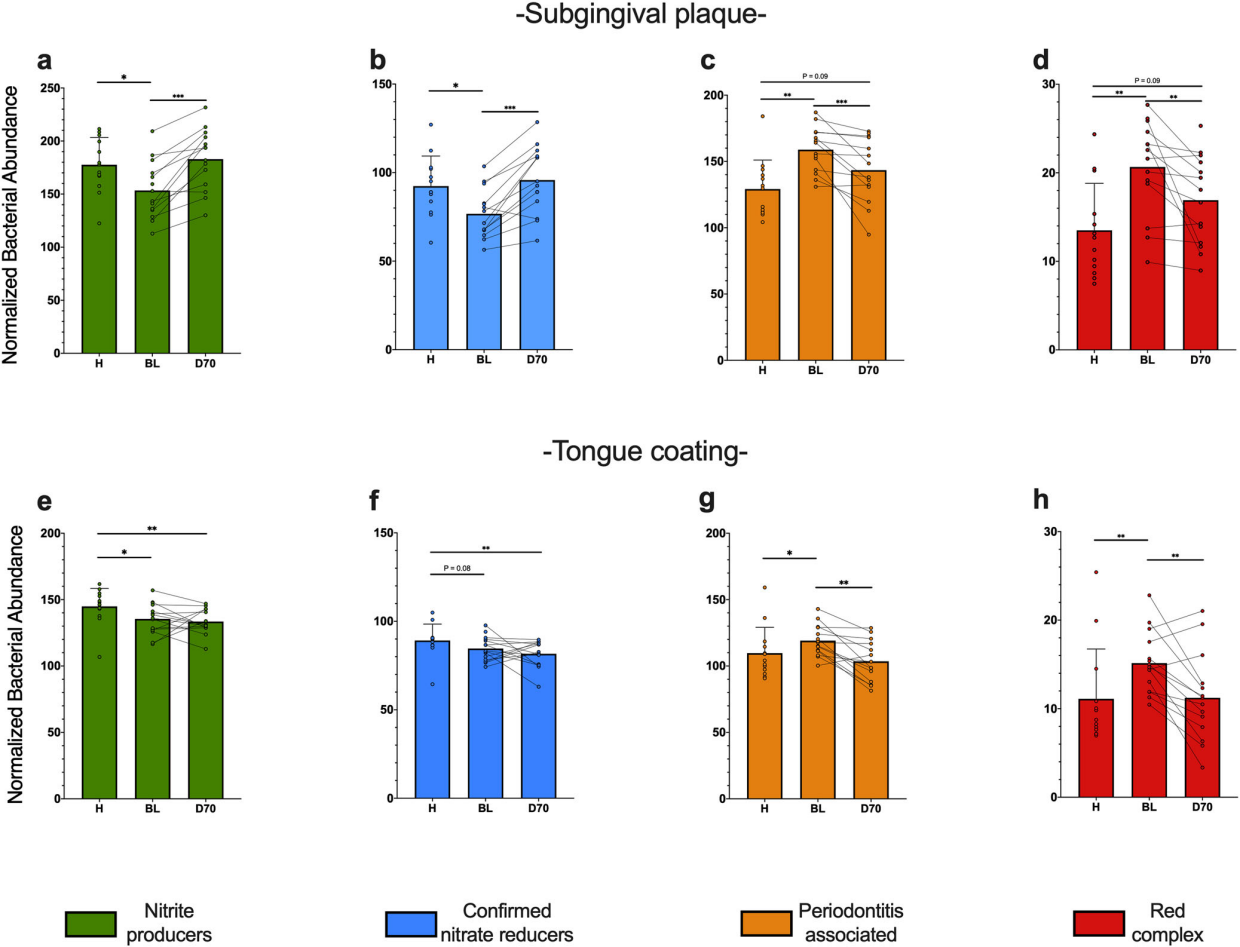
Changes in nitrate-reducing bacteria and periodontitis-associated species. Bar graphs show the transformed abundance of bacterial groups in subgingival plaque (**a**–**d**) and tongue samples (**e**–**h**), as estimated by high-throughput sequencing of the 16 S rRNA gene. The relative abundances of species were transformed with ANCOM-BC and grouped in nitrite-producers (**a**, **e**), confirmed nitrate reducers (**b**, **f**), periodontitis-associated species (**c**, **g**) and red complex (**d**, **h**) as done previously^5^. *p < 0.05, **p < 0.01, ***p < 0.001 according to a Wilcoxon test (unpaired comparisons) o Mann-Whitney-U test (paired comparison).

In periodontitis, the median of relative abundances (Fig. 5) of nitrite-producers, confirmed nitrate-reducers, periodontitis-associated species, and red complex were, 15.32%, 2.53%, 25.24% and 3.03% in subgingival plaque, respectively, and 22.52%, 16.99%, 8.79% and 0.12% in tongue samples. In periodontal health, these were 21.45%, 6.36%, 8.87% and 0.06% in subgingival plaque and 24.52%, 15.68%, 4.76% and 0.01% in tongue communities, respectively. Significant differences between groups were determined after ANCOM-BC standardization of compositional data (Fig. 7). In subgingival plaque, all periodontitis-associated bacteria (Fig. 7c), including the red complex (Fig. 7d), were higher in periodontitis compared with periodontal health (*p* < 0.01) and decreased after periodontal treatment (*p* < 0.01) as expected. Interestingly, the same pattern was found in tongue samples (Fig. 7g, h), indicating that this habitat is also affected by periodontitis. For nitrite-producing species, including confirmed nitrate-reducing species, differences were found between periodontitis and periodontal health in both subgingival (Fig. 7a, b) and tongue samples (Fig. 7e, f) (all *p* < 0.5). However, after periodontal treatment, there was a clear increase in these groups in subgingival plaque (*p* < 0.001 for nitrite-producers and confirmed nitrate-reducers), but not in tongue samples.

#### Correlations between bacteria in subgingival plaque and tongue samples

To evaluate the possible relationship between the abundance of bacteria in plaque and tongue samples, a correlation analysis was performed (Table 3). In total, significant correlations were found between 20 taxa. When grouping all samples (*n* = 41), some of these correlations corresponded to known periodontitis-associated species, including *Eubacterium saphenum* (r = 0.59, *p* < 0.05) and *Prevotella nigrescens* (r = 0.51, *p* < 0.05), while others were known nitrite-producing species, including *Haemophilus parainfluenzae* (r = 0.66, *p* < 0.001), *Rothia dentocariosa* (r = 0.65, *p* < 0.001), *Rothia mucilaginosa* (r = 0.58, *p* < 0.05), *Veillonella atypica* (*r* = 0.54, *p* < 0.05), *Corynebacterium durum* (r = 0.53, *p* < 0.05) and *Actinomyces naeslundii* (r = 0.43, *p* < 0.05).

**Table 3:** Bacteria correlations between subgingival plaque and tongue samples

	Species	Subgingival plaque (%) Median (Q1,Q3)	Tongue coating (%) Median (Q1,Q3)	Spearman	Adjusted *p* value
All groups (*n* = 41)	*Haemophilus NA*	0.56 (0.14,2.60)	4.54 (2.14,7.59)	0.74	< 0.0001
	*Leptotrichia hofstadii*	0.02 (0.00,0.18)	0.01 (0.00,0.04)	0.71	< 0.0001
	*Haemophilus parainfluenzae*	0.02 (0.00,0.28)	0.072 (0.17,1.25)	0.66	< 0.001
	*Rothia dentocariosa*	0.46 (0.05,2.58)	0.06 (0.03,0.13)	0.65	< 0.001
	*Streptococcus NA*	7.46 (5.41,12.99)	21.74 (17.07,28.63)	0.61	< 0.01
	*Eubacterium saphenum*	0.00 (0.00,0.07)	0.00 (0.00,0.03)	0.59	< 0.05
	*Rothia mucilaginosa*	0.06 (0.03,0.11)	4.08 (2.71,8.35)	0.58	< 0.05
	*Cardiobacterium hominis*	0.45 (0.09,1.15)	0.01 (0.01,0.02)	0.57	< 0.05
	*Veillonella atypica*	0.03 (0.02,0.19)	1.39 (0.42,3.32)	0.54	< 0.05
	*Corynebacterium durum*	0.43 (0.04,1.33)	0.02 (0.01,0.06)	0.53	< 0.05
	*Bergeyella NA*	0.22 (0.05,0.51)	0.10 (0.03,0.24)	0.52	< 0.05
	*Gemella morbillorum*	0.46 (0.10,0.69)	0.03 (0.02,0.08)	0.51	< 0.05
	*Prevotella nigrescens*	1.03 (0.10,2.41)	0.07 (0.02,0.15)	0.51	< 0.05
	*Actinomyces naeslundii*	0.82 (0.39,1.50)	0.04 (0.01,0.09)	0.43	< 0.05
Periodontitis BL + D70 (*n* = 29)	*Porphyromonas gingivalis*	0.03 (0.00,1.74)	0.04 (0.00,0.14)	0.73	< 0.05
	*Haemophilus NA*	0.36 (0.09,1.10)	3.34 (1.78,5.46)	0.72	< 0.05
	*Filifactor alocis*	0.23 (0.01,0.50)	0.02 (0.00,0.07)	0.68	< 0.05
	*Leptotrichia wadei*	0.60 (0.20,2.57)	0.05 (0.03,0.16)	0.69	< 0.05
	*Veillonella dispar*	0.02 (0.02,0.04)	4.46 (2.18,6.83)	0.68	< 0.05
	*Streptococcus NA*	7.20 (5.52,10.88)	21.49 (16.20,28.72)	0.65	< 0.05
Periodontitis BL (*n* = 14)	*Eubacterium saphenum*	0.05 (0.00,0.26)	0.00 (0.00,0.01)	0.86	< 0.05

Significant correlations between bacteria and several clinical parameters were also observed. As expected, periodontitis-associated species in subgingival plaque (e.g. *Prevotella intermedia, Peptostreptococcus stomatis*, unclassified *Fretibacterium*) correlated positively with BoP and PoC, while health-associated species (e.g., *Rothia dentocariosa, Veillonella dispar, Kingella oralis*) correlated negatively with these parameters (Supplementary Table 1). Interestingly, significant correlations were also found between tongue bacteria and periodontal parameters. For example, nitrate-reducing bacteria (e.g., *Actinomyces massiliensis* and *Rothia dentocariosa*) correlated negatively with BoP (Supplementary Table 2).

Another interesting result was the positive correlation of *Tannerella forsythia* in tongue coating with systolic and diastolic blood pressure (r = 0.42 and 0.50, respectively, both *p* < 0.05), while the known nitrite-producing *Corynebacterium durum* correlated negatively with diastolic blood pressure (r = -0.44, *p* < 0.05) (Supplementary Table 2). Unclassified *Kingella spp*. (representatives of a known nitrate-reducing genera) in subgingival plaque and tongue coating also correlated negatively with diastolic blood pressure (r = -0.42-0.43, both *p* < 0.05) (Supplementary Tables 1 and 2).

## Discussion

Periodontitis is a common oral disease that increases the risk of different systemic conditions such as cardiovascular diseases, diabetes and preeclampsia^38^. Interestingly, a closer look at these systemic complications reveals that they are associated with a deficit in nitric oxide availability^39–41^. This is very relevant because the link between periodontitis and systemic diseases has been historically associated with the pro-inflammatory effect of periodontal pathogens^42^. However, recent data indicate that periodontal disease is also associated with a decrease in the NRC of the oral microbiota^5^, which is an essential function in the nitrate-nitrite-nitric oxide pathway^13^. Therefore, it would be feasible that the oral-systemic connection is not only derived from a detrimental impact of periodontal pathogens, but also from a diminished effect of beneficial microbes’ activity in the oral cavity. In this study we found that a well-documented example of systemic effect derived from nitrate reduction, i.e., the blood-pressure lowering effect of nitrate-rich beetroot juice, is impaired in periodontitis. However, after periodontal treatment (i.e., PMPR, oral hygiene instruction, and subgingival instrumentation), this blood-pressure lowering effect was recovered. These results were accompanied by lower levels of nitrate-reducing bacteria in tongue and subgingival plaque samples of patients with periodontitis, which increased only in subgingival plaque after periodontal treatment. Future studies should determine if a decrease in the NRC of the oral microbiota in periodontitis could contribute to an increased risk of hypertension or other systemic complications.

To test the effect of periodontitis on the nitrate-nitrite-nitric oxide pathway, beetroot juice was consumed by 15 periodontitis patients before (baseline) and 70 days after periodontal treatment, as well as by 15 healthy individuals. Nitrate from beetroot juice is reduced to nitrite by oral bacteria^6^, increasing the salivary nitrite levels as observed in our study. It is known from many other studies^12,29^ that this leads to an increase in plasma nitrite, resulting in higher nitric oxide availability and increased vasodilation that can decrease blood pressure. While beetroot juice has other components like antioxidants and polyphenols, it is known that nitrate-depleted beetroot juice does not have blood pressure-lowering effects, while nitrate-salts by themselves do^29,43,44^. In our study, one and a half hour after beetroot juice intake, there was a significant blood-pressure lowering effect in healthy individuals (a decrease of 6.68 ± 6.02 mmHg in SBP and 3.52 ± 4.45 mmHg in DBP, *p* < 0.01), in agreement with previous reports^29^. However, this blood-pressure lowering effect was not observed in individuals with periodontitis at baseline. After periodontal treatment, the blood-pressure lowering effect was recovered (a decrease of 4.36 ± 7.59 mmHg in SBP and 3.72 ± 4.47 mmHg in DBP, *p* < 0.05 and *p* < 0.01, respectively). Apart from vasodilation and blood pressure-lowering effects, this nitrate-derived nitric oxide can lead to other benefits, including improved endothelial function, increased sport performance and potential antidiabetic effects^8^. The possibility of periodontitis limiting the benefits of dietary nitrate should be confirmed in future randomized controlled trials.

Interestingly, periodontitis increases the risk of different systemic conditions associated with a deficit in nitric oxide availability^39–41^, which appear to benefit from nitrate intake^8,29,43,45^. For example, periodontitis increases the risk of high blood pressure and diabetes^46,47^ both associated with a deficit in nitric oxide availability. On the one hand, sterilizing the oral cavity with an antiseptic mouthwash leads to an acute increase in blood pressure due to the loss of nitrate-reducing bacteria^13^, while mouthwash usage correlated with diabetes development^48^. On the other hand, stimulating nitrate-reducing bacteria by nitrate intake has blood pressure lowering and potential anti-diabetic effects^8^. In our study, we evaluated if the loss of the blood pressure-lowering effect of nitrate was accompanied by a decrease in the levels of nitrate-reducing bacteria in subgingival plaque and tongue samples.

In subgingival plaque, we found that nitrate-reducing bacteria (including *Kingella oralis*, *Actinomyces* spp. and *Rothia dentocariosa*) were associated with periodontal health, which is in line with previous results^5^. Apart from producing nitrite, some of these bacteria have genes to produce nitric oxide^4^. In agreement with the subgingival plaque results, nitrate-reducing bacteria in tongue samples (including *Veillonella* spp. and *Rothia dentocariosa*) were more abundant in periodontal health. It is thought that the tongue community has a main role in the nitrate-nitrite-nitric oxide pathway as revealed by measures of nitrate reduction capacity in different oral tissues^17^. However, the recovery of the blood pressure-lowering effect of beetroot juice after periodontal treatment was associated with an increase in nitrate-reducing bacteria in subgingival plaque (the community that was treated), but not in the tongue coating. Additionally, a recent study linked the nitrite production capacity of subgingival plaque to cardiovascular health^49^. The potential impact of nitrite and nitric oxide produced by subgingival bacteria on blood pressure and cardiovascular health warrants further investigation, such as by testing the effect of a subgingival gel containing nitrate.

Notably, 1.5 h after taking an identical nitrate dose, more salivary nitrate was found in samples of periodontitis patients (893 mg/L = 14.40 mM) compared to samples of healthy individuals (616 mg/L = 9.94 mM). However, after periodontal treatment, the post-BRJ salivary nitrate levels did not differ significantly from health. These results indicate that the healthy oral microbiota could reduce more nitrate than the oral microbiota in untreated periodontitis. Similarly, there were no differences in salivary nitrite between health and periodontitis. If more nitrate was reduced, but equal amounts of nitrite were detected, it appears that part of the produced nitrite was converted into other compounds (e.g., nitric oxide). This could explain the difference in blood-pressure lowering effects of beetroot juice between periodontal health and periodontitis. These results are in line with data by Rosier et al. ^5^ that incubated saliva with 8 mM nitrate and found that significantly more nitrate was reduced by healthy samples compared to periodontitis samples^5^. Additionally, they found that after periodontal treatment, the nitrate reduction capacity was recovered to healthy levels.

In the current study, the periodontal treatment significantly led to an improvement in all periodontal parameters, showing that inflammation was reduced 70 days after treatment. Apart from an increase in health-associated nitrate-reducing species in subgingival plaque, there was a decrease in periodontitis-associated species (including *Tannerella forsythia* and unclassified *Fretibacterium*). Interestingly, periodontitis-associated species also decreased in tongue samples after periodontal treatment, including *Porphyromonas gingivalis* and *Filifactor alocis*, which belong to the most disease-associated species^35,50^, but no significant increases in nitrate-reducing species were found in this habitat. The ACE and Chao1 alpha-diversity indexes showed that there were more species in subgingival plaque of periodontitis patients compared with healthy individuals, which is known from other studies^50,51^, but surprisingly, this was also observed in tongue samples. Similarly, CCA and ADONIS analyses revealed that the tongue coating is different in periodontitis compared to health. In contrast, the periodontal treatment in periodontitis patients led to significant differences in alpha- and beta-diversity in subgingival plaque as previously demonstrated^16^, but not in tongue coating. It should be noted that the periodontal treatment in this study included plaque removal and subgingival instrumentation, when necessary, but not tongue cleaning.

It has been proposed that tongue coating can act as a reservoir for periodontitis-associated bacteria, as most periodontal pathogens are detected in the tongue^52^ and higher tongue levels of *Porphyromonas gingivalis* were found in periodontitis^53,54^. Periodontitis is also associated to plaque accumulation due to a lack of oral hygiene, while tongue coating is also found to be more abundant in this oral disease^53^. In our study, clinically relevant bacteria, including periodontitis associated species (e.g., *Eubacterium saphenum* and *Prevotella nigrescens*) and nitrate-reducers (e.g., *Rothia dentocariosa*, *Rothia mucilaginosa*, *Veillonella atypica* and *Actinomyces naeslundii*), correlated between tongue and subgingival plaque samples, supporting the idea that the tongue could act as a bacterial reservoir for plaque communities and vice versa. In summary, our data indicate that periodontal treatment may not only reduce periopathogens in subgingival plaque but also in tongue coating. This could potentially slow down the recolonization of periodontitis-associated bacteria at the gums and future studies should explore the effect of tongue cleaning on periodontal treatment efficiency. Related to this, tongue cleaning has previously been associated with higher levels of nitrate-reducing bacteria and more efficient nitrate reduction^55^. This suggests that adding tongue cleaning to standard periodontal treatment could further increase periodontal health-associated nitrate-reducing bacteria on the tongue surface. Specifically, the nitrite and nitric oxide produced by tongue bacteria could increase in saliva, which can reduce the numbers of periopathogens^7^ and improve cardiometabolic parameters^6,8^. In light of this, we found a negative correlation between nitrate-reducing bacteria on the tongue and bleeding on probing (*Actinomyces massiliensis* and *Rothia dentocariosa*) and blood pressure (*Corynebacterium durum* and unclassified *Kingella*). The relationship between tongue cleaning, nitrate metabolism and blood pressure should therefore be further explored.

In our study, it was found that the salivary flow increased 1.5 h after beetroot juice intake, especially in healthy individuals. However, a control group that ingested the same amount of nitrate-free water was not included, and it is therefore possible that part of this effect derives from hydration^56^. Salivary flow is instrumental for oral health, as saliva contributes to pH buffering and food clearance, in addition to containing antibodies, antimicrobial peptides and enzymes that contribute to oral and systemic homeostasis. In fact, reduced salivary flow, either derived from certain health conditions or from use of certain medicaments, results in oral health complications^57^. Nitric oxide could play a role in salivary secretion through the non-adrenergic, non-cholinergic pathways of the autonomic nervous system^58^. In addition, in animal models, dietary nitrate supplementation has been shown to prevent radiotherapy-induced xerostomia^59^. Thus, the potential effect of the oral microbiome in the regulation of salivary secretion through nitrate metabolism should be studied in the future.

### Study limitations

A main limitation of this study was that the population size was limited to 15 individuals per group (periodontal health, periodontitis BL and periodontitis D70). Additionally, for the microbiota analysis, the final sample size for both tongue and subgingival samples was 13–14 per group (healthy individuals or periodontitis patients at BL and D70) because several samples were lost during the DNA isolation and sequencing procedure. Although this number is small, previous studies have shown that comparing 10 healthy individuals with 10 periodontitis patients^60^, as well as 11 periodontitis patients before and after treatment^61^ can yield significant results. Similarly, multiple significant differences were found when comparing the groups in our study, providing relevant new insights that should be confirmed in larger populations and across different ethnicities. In our study, we obtained evidence that periodontitis could limit the blood pressure-lowering effect of beetroot juice by interfering with the nitrate-reducing oral microbiota. Importantly, we demonstrated that multiple factors known to affect blood pressure (e.g., weight, BMI and relevant dietary factors) were not significantly different between the periodontitis and healthy groups at baseline, and it is known that, without a specific intervention, these factors are unlikely to change over 70 days. Nevertheless, our results should be confirmed in a randomized controlled trial that takes into account potential confounding factors such as anxiety associated with the clinic or dentist, as well as physical exercise and other individual habits that may change over time. In such a study, the effect of a nitrate-rich supplement should be compared with a placebo supplement. Additionally, the healthy individuals could receive a standard dental cleaning and come back to the clinic 70 days after this, proving two timepoints for both groups of individuals.

### Concluding remarks

To our knowledge, this is the first study that provides evidence that in periodontitis, the systemic benefits of nitrate-reduction by the oral microbiota could be impaired. Specifically, we showed that the blood-pressure lowering effect of nitrate-rich food was impaired in periodontitis but recovered after periodontal treatment. It is known that the nitrate-derived nitric oxide, which is responsible for the blood-pressure lowering effects, leads to different other cardiovascular and metabolic benefits^6,8^. Our results therefore indicate that the potential loss of nitrate-reduction in periodontitis could contribute to the development of systemic complications associated with a deficit in nitric oxide availability (e.g., cardiovascular diseases, diabetes and pre-eclampsia, among others)^38–41^. However, this hypothesis should be confirmed in randomized controlled trials accounting for cofounding factors. Nitrate-reducing species, including *Rothia* spp., *Veillonella* spp. and/or *Actinomyces* spp., in tongue and subgingival plaque samples were found in lower levels in periodontitis. Therefore, the probiotic potential of these bacteria, as well as the prebiotic potential of nitrate, to treat periodontitis and comorbidities should be explored in future studies. Periodontal treatment led to a decrease in levels of periodontitis-associated species in subgingival and tongue samples, but only increased nitrate-reducing species in subgingival samples. Future studies should test if tongue cleaning can further stimulate de nitrate-nitrite-nitric oxide pathway and improve treatment outcomes in periodontitis patients.

## Supplementary information

Supplementary Figures and Tables

Supplementary Datasheet

## Data Availability

The subgingival and tongue sequencing reads of the periodontitis patients before (B.L.) and after (D70) treatment, and the healthy individuals have been deposited in the NCBI Sequencing Read Archive (SRA) with project title. “The blood pressure lowering effect of beetroot juice is impaired in periodontitis and recovered after periodontal treatment” (BioProject ID PRJNA1200033). Any further data is available upon reasonable request from the corresponding author.
