# Supplementary material for: The blood pressure lowering effect of beetroot juice is impaired in periodontitis and recovered after periodontal treatment
Source: NPJ Biofilms Microbiomes. 2025 Jan 9;11:10. doi: 10.1038/s41522-024-00622-5 (PMC11717912; doi:10.1038/s41522-024-00622-5)
Supplement: Supplementary file 1 — Supplementary Figures and Tables [file 41522_2024_622_MOESM1_ESM.pdf]

The blood pressure lowering effect of beetroot juice is impaired in periodontitis and recovered after periodontal treatment

Nydia Y. Sanchez-Orozco[1*], Bob T. Rosier[2*#], Alondra Ruiz-Gutierrez[3], Fabiola Marquez-Sandoval[1], Alejandro Artacho[2], Lucrecia Carrera-Quintanar[1#], Alex Mira[2,4]

[1] PhD Program in Translational Nutrition Sciences, Department of Human Reproduction, Child Growth and Development, University Center of Health Sciences (CUCS), University of Guadalajara (UdeG), Guadalajara, Jalisco, Mexico.
[2] Department of Health and Genomics, FISABIO Foundation, Valencia, Spain.
[3] Specialty of Periodontics, Department of Integral Dental Clinics, University Center of Health Sciences (CUCS), University of Guadalajara (UdeG), Guadalajara, Jalisco, Mexico.
[4] CIBER Center for Epidemiology and Public Health (CIBER-ESP), Madrid, Spain.

*These authors contributed equally: Nydia Y. Sanchez-Orozco and Bob T. Rosier

[#]Correspondance: Bob T. Rosier (bob.rosier@fisabio.es) and Lucrecia Carrera-Quintanar (lucrecia.carrera@academicos.udg.mx)

Senior author: Alex Mira

Supplementary Information (2 tables, 2 figures)

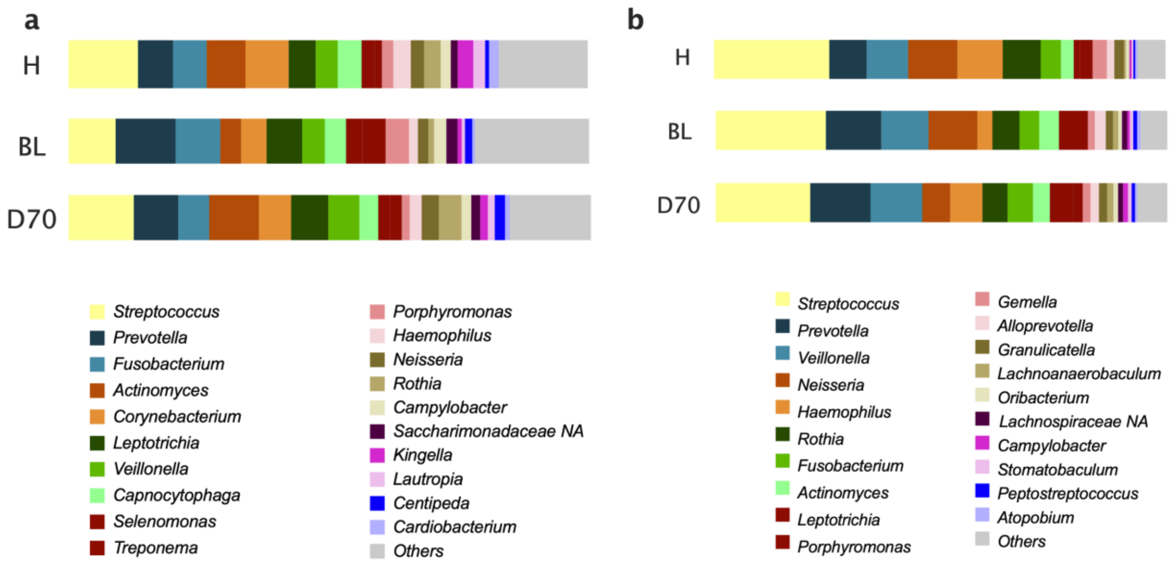

Supplementary Figure 1. Relative bacterial abundances in subgingival plaque (A) and tongue coating (B). Bar graphs show the top 30 most abundant genera in healthy individuals (H) and periodontitis patients before (BL) and after periodontal treatment (D70). Relative abundances are shown.

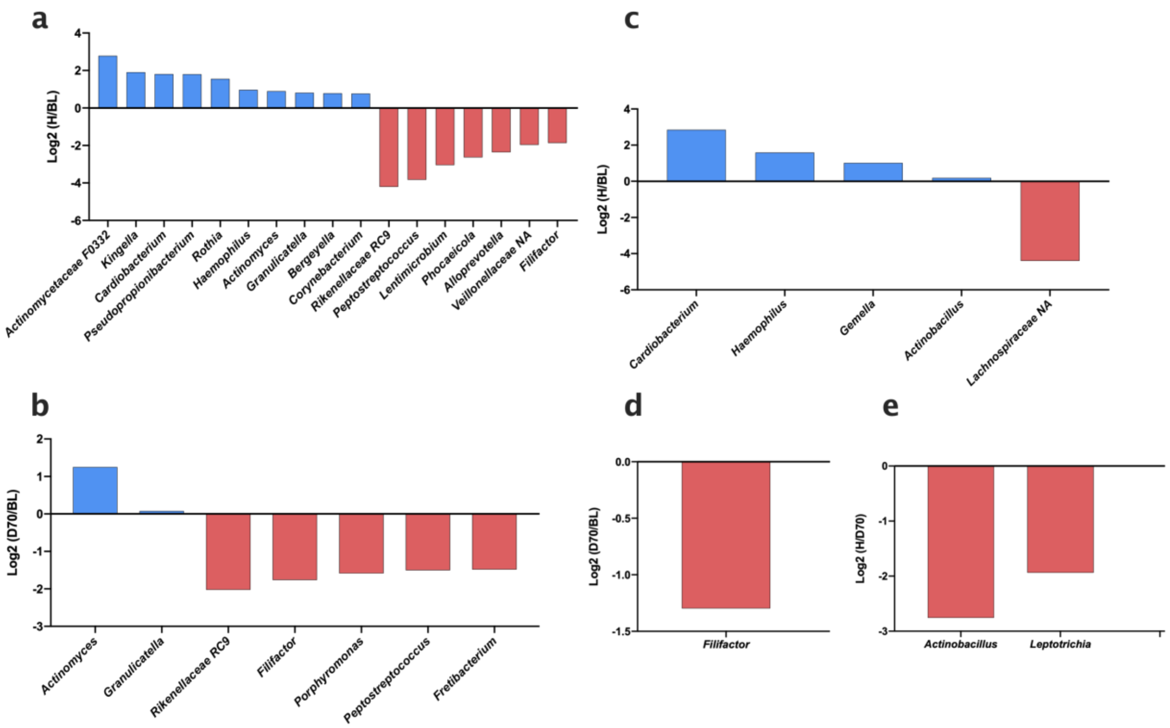

Supplementary Figure 2. Log$_2$ fold change of all significantly different genera in subgingival plaque (A, B) and tongue (C-E). Bar graphs show comparisons between healthy individuals (H) and periodontitis patients at baseline (BL) and 70 days after treatment (D70). The groups that are compared are H and BL (A), and D70 and BL (B) in subgingival plaque, as well as H and BL (C), D70 and BL (D), and H and D70 (E) in tongue coating. Blue bars (positive values) indicate genera which increased significantly after treatment or are more abundant in health; red bars (negative values) indicate genera which significantly decreased after treatment or are more abundant in disease. To calculate the ratios, relative abundances were used. All plotted organisms were significantly different (adjusted p-values < 0.05) between two groups after standardization of the compositional data by ANCOM-BC. Organisms marked with NA indicate that they could not be assigned at the genus level.

Supplementary Table 1. Spearman-Rho correlation between relative abundance of subgingival plaque species with clinical parameters. Displaying p(adjusted)<0.05, data displayed as Spearman R (adjusted p-value)

	BoP	PD	CAL	PoC	SBP	DBP
Gemella NA	-0.56 (p<0.05)	-	-		-	-
Rothia dentocariosa	-0.56 (p<0.05)	-	-	-0.57 (p<0.05)	-	-
Kingella oralis	-0.54 (p<0.05)	-	-	-0.52 (p<0.05)	-	-
Phocaeicola abscessus	0.52 (p<0.05)	-	-	0.53 (p<0.05)	-	-
Prevotella intermedia	0.51 (p<0.05)	-	-	0.57 (p<0.05)	-	-
Treponema maltophilum	0.52 (p<0.05)	-	-	-	-	-
Cardiobacterium hominis	-	-0.50 (p<0.05)	-0.55 (p<0.05)	-0.52 (p<0.05)	-	-
Actinomycetaceae F0332 NA	-	-	-	-0.61 (p<0.01)	-	-
Actinomyces gerencseriae	-	-	-	-0.58 (p<0.05)	-	-
Fretibacterium NA	-	-	-	0.57 (p<0.05)	-	-
Peptostreptococcus stomatis	-	-	-	0.56 (p<0.05)	-	-
Veillonellaceae NA	-	-	-	0.50 (p<0.05)	-	-
Leptotrichia hongkongensis	-	-	-	-0.49 (p<0.05)	-	-
Veillonella dispar	-	-	-	-0.49 (p<0.05)	-	-
Granulicatella NA	-	-	-	-0.49 (p<0.05)	-	-
Veillonella atypica	-	-	-	-	0.48 (p<0.05)	
Desulfobulbus NA	-	-	-	-	0.47 (p<0.05)	0.57 (p<0.01)
TM7x NA	-	-	-	-	-0.41 (p<0.05)	-
Kingella NA	-	-	-	-	-	-0.43 (p<0.05)

Supplementary Table 2. Spearman-Rho correlation between relative abundance of tongue species with clinical parameters. Displaying p(adjusted)<0.05, data displayed as Spearman R (adjusted p-value).

	BoP	PD	CAL	POC	SBP	DBP
Rothia dentocariosa	-0.63 (p<0.01)	-	-	-	-	-
Actinomyces massiliensis	-0.62 (p<0.01)	-	-	-	-	-
Lautropia mirabilis	-0.54 (p<0.05)	-	-0.58 (p<0.05)	-	-	-
Actinobacillus pleuropneumoniae	-0.54 (p<0.05)	-	-0.68 (p<0.01)	-	-	-
Streptococcus NA	-0.51 (p<0.05)	-	-	-	-	-
Veillonella NA	-0.51 (p<0.05)	-	-	-0.49 (p<0.05	-	-
Kingella oralis	-0.51 (p<0.05)	-	-	-	-	-
Actinobacillus pleuropneumoniae	-	-0.52 (p<0.05)	-	-0.60 (p<0.05)	-	-
Veillonella rogosae	-	-0.50 (p<0.05)	-0.52 (p<0.05)	-	-	-
Veillonella NA	-	-0.49 (p<0.05)	-0.59 (p<0.05)	-	-	-
Gemella sanguinis	-	-	-0.58 (p<0.05)	-0.49 (p<0.05	-	-
Veillonella massiliensis	-	-	-0.53 (p<0.05)		-	-
Haemophilus NA	-	-	-0.53 (p<0.05)	-0.55 (p<0.05)	-	-
Bergeyella NA	-	-	-0.51 (p<0.05)	-	-	-
Cardiobacterium hominis	-	-	-0.52 (p<0.05)	-	-	-
Actinomycetaceae F0332 NA	-	-	-	-0.49 (p<0.05	-	-
Leptotrichia wadei	-	-	-	-	0.48 (p<0.05)	-
Veillonellaceae NA	-	-	-	-	0.43 (p<0.05)	0.47 (p<0.05)
Tannerella forsythia	-	-	-	-	0.42 (p<0.05)	0.50 (p<0.05)
Corynebacterium durum	-	-	-	-	-	-0.44 (p<0.05)
Kingella NA	-	-	-	-	-	-0.42 (p<0.05)
